# IMPLICATIONS OF THE COVID-19 PANDEMIC ON ADULT DAY SERVICES

**DOI:** 10.1093/geroni/igad104.2668

**Published:** 2023-12-21

**Authors:** Katherine Marx, Lauren Parker, Laura Gitlin

**Affiliations:** Johns Hopkins University, Baltimore, Maryland, United States; Johns Hopkins University, Baltimore, Maryland, United States; Drexel University, Philadelphia, Pennsylvania, United States

## Abstract

Covid-19 hit Adult Day Services (ADS) hard with sites across the country forced to close. As part of the ADS-Plus study, two focus groups were conducted to understand the impact of Covid-19 on ADS sites, their participants, and staff. Six sites across the country, represented by nine staff and administration (females=n, males=1) participated in focus groups. Focus groups were recorded, transcribed verbatim, and coded by the authors. Four themes emerged, (1) Ways to stay in touch with families; (2) Issues reopening; (3) Family concerns; and (4) Future of the ADS Industry. Sites stayed in touch with families through a variety of measures, including virtual daycare or activities via zoom, house calls, and help obtaining food. Upon reopening, there were multiple issues with getting the census back for both participants and staff. Families were reluctant to return due to fear of Covid-19, placing their family member in LTC due to decline in functioning or due to work, and using more home services. Staff were reluctant to return due to fear of Covid-19 and salary, some reporting they made more on unemployment. There was a decline in both physical and cognitive health of ADS participants, attributed to the lack of socialization. In looking towards the future, focus group participants felt that the old approach of doing ADS is not sustainable and that the industry needs to expand to include more home options. Also, funding of ADS needs to be addressed as many facilities are not able to keep up with increasing salaries.

